# Associations between lifestyle behaviour changes and the optimal well-being of middle-aged Japanese individuals

**DOI:** 10.1186/s13030-021-00210-5

**Published:** 2021-04-01

**Authors:** Toshihiro Takao, Naoki Sumi, Yoshiyuki Yamanaka, Sohachi Fujimoto, Tomoari Kamada

**Affiliations:** Department of Health Care Medicine, Kawasaki Medical School, 577 Matsushima, Kurashiki, 701-0192 Japan

**Keywords:** Annual health check-up, Dietary habits, Physical activity, World Health Organization-five well-being index

## Abstract

**Background:**

Psychological well-being has been associated with reduced mortality rates in both healthy and diseased populations. However, there is considerably less evidence on the effect of lifestyle behaviours on positive health outcomes such as well-being. This study examines the association between lifestyle behaviours and optimal well-being.

**Methods:**

From a total of 4324 Japanese individuals who participated in an annual health check-up in 2017, this study recruited 2295 participants (mean age: 49.3 ± 8.4 years; female: 54.3%) without a history of cerebrovascular, cardiovascular, or chronic renal disease and not on medication for hypertension, diabetes, or dyslipidaemia. The World Health Organization-Five Well-Being Index (WHO-5) scores were compared to self-reported scores on each of the following items: dietary habits, physical activity, smoking, alcohol consumption, and sleep quality. Logistic regression analysis was used to examine the association between optimal well-being (the top quartile of WHO-5 scores) and individual lifestyle behaviours. The association between change in dietary habits and physical activity from 2016 to 2017 and optimal well-being was also investigated.

**Results:**

Good dietary habits and regular physical activity were associated with higher raw WHO-5 scores and were positively associated with optimal well-being after adjusting for age, sex, body mass index (BMI), and sleep quality. Raw WHO-5 scores were significantly higher in those who maintained good dietary and physical activity behaviours than in those who did not. Furthermore, maintaining regular physical activity for two years was positively associated with optimal well-being, after adjusting for age, sex, BMI, and sleep quality.

**Conclusion:**

These results demonstrate that not only currently practising good dietary and physical activity behaviours but also maintaining such behaviours over time is associated with optimal well-being. Maintaining good lifestyle behaviours, particularly regarding physical activity, could potentially improve people’s well-being.

## Background

Psychological well-being is an important part of overall well-being and has been associated with reduced mortality rates in both healthy and diseased populations [1]. Additionally, lifestyle behaviours such as smoking, alcohol consumption, diet, physical activity, sitting time and sleep duration and quality have been associated with mortality [2–5]. Within lifestyle behaviours, particularly becoming more sedentary and a poor diet may affect our health condition. We previously reported that the Patient Health Questionnaire (PHQ)-9 scores in the participants who never ate breakfast were higher than those who ate breakfast every day [6], suggesting that these lifestyle behaviours may be associated with psychological distress.

In a more specific example, lower consumption of fruits and vegetables and higher consumption of French fries, fast food, soda, and variance-adjusted daily teaspoons of sugar were associated with moderate or serious psychological distress [7]. Additionally, older women who spent more time watching TV than engaging in other sedentary behaviours showed a higher number of depressive symptoms; thus, increasing recreational physical activity may improve mental health in older adults, particularly among women [8], suggesting that poor dietary and physical activity behaviours seriously affect mental health.

However, relative to these negative health outcomes, there is considerably less evidence on the effect of lifestyle behaviours, such as dietary and physical activity, on positive health outcomes. Prendergast and colleagues reported the association with optimal well-being was greater for those who reported more exercising and less sitting [9]. In addition, Piqueras et al. showed people self-reporting daily physical activity and having lunch, fruits, and vegetables each day had a higher likelihood of being classified as ‘very happy’ [10].

Numerous self-report screening instruments for depression exist, including the PHQ-9 and the General Health Questionnaire-30. However, such psychometric instruments are more useful in clinical practice [11]. In contrast, the 5-item World Health Organization Well-Being Index (WHO-5) exemplifies a positively worded questionnaire [12]. A recent study [13] examined the associations between multiple lifestyle behaviours and optimal well-being, classifying the top quintile of WHO-5 scores as optimal well-being and the remaining quintiles as non-optimal well-being. Good dietary habits, physical activity, sitting time, sleep duration, and higher sleep quality were positively associated with optimal well-being [13].

Subjective well-being and health are closely linked to age [14]. A U-shaped relation between evaluative well-being and age was found in high-income, English-speaking countries, with the lowest levels of well-being reported in middle-aged individuals [14, 15]; however, this pattern was not universal [16]. Considering the above evidence, middle-aged individuals seem to be particularly vulnerable to experiencing a low level of well-being and mental distress. Nonetheless, many previous studies have focused on well-being in older adults [17, 18], not that of middle-aged individuals. To the best of our knowledge, there is no study investigating the association between lifestyle and well-being using the WHO-5 score in a Japanese primary care population**.** The present study focuses on the association between lifestyle behaviours such as dietary and physical activity and optimal well-being in middle-aged Japanese individuals. Moreover, it examines the effects of changes in dietary and physical activity behaviours on optimal well-being.

## Materials and methods

### Study population

**Fig. 1:**
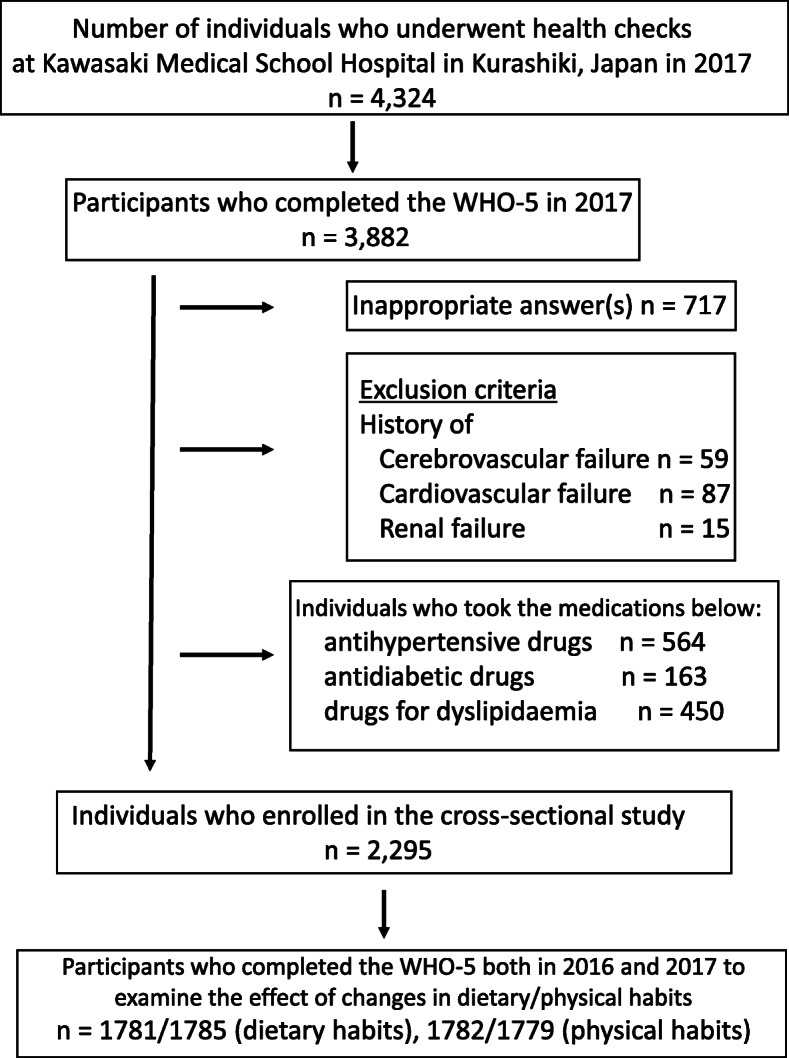
Flowchart of participant recruitment

Data from 4324 individuals who visited the Kawasaki Medical School Hospital (Kurashiki, Japan) for an annual health check-up in 2017 were examined. The participants were mostly individuals who wished to undergo a medical examination and employees for whom an annual health check-up was mandated by law. Of them, this study enrolled 2295 individuals without a history of cerebrovascular, cardiovascular, or chronic renal disease, which may affect well-being, and individuals not taking medication for hypertension, diabetes, or dyslipidaemia. However, the number of respondents for each question described below varied depending on the question items. Moreover, participants whose WHO-5 scores for both 2016 and 2017 were measured were recruited to examine the association between change in dietary/physical activity behaviours and optimal well-being (Fig. 1).

### Ethical considerations

Because patient-identifying data such as name and date of birth from the healthcare database were not obtained, the need for informed consent was waived. The survey was anonymous. However, we informed potential participants who visited the hospital for an annual health check-up during the study’s target period. They were given the opportunity to opt out of the survey, and we took steps to delete personal data if they did not wish to participate in the research. The study protocol followed the Japanese Government’s Ethical Guidelines Regarding Epidemiological Studies in accordance with the Declaration of Helsinki. This study was approved by the Kawasaki Medical School Ethics Committee (Approval No. 2632–4). Personal information of potential participants was keyed into a secure database maintained by an exclusive information manager.

### Measurement of body mass index and obesity classification

BMI was calculated based on weight and height measurements, employing the following equation: weight (kg) divided by the square of height (m). Participants were classified as either underweight (BMI < 18.5), normal weight (18.5 ≤ BMI < 25), overweight (25 ≤ BMI < 30), or obese (30 ≤ BMI).

### The 5-item World Health Organization well-being index (WHO-5)

The WHO-5 is among the most widely used questionnaires for the assessment of subjective psychological well-being. Since its first publication in 1998, the WHO-5 has been translated into more than 30 languages and has been used in research studies worldwide [19]. The validity of the WHO-5 has been reported in many countries [20, 21]. The WHO-5 items are: ‘I have felt cheerful and in good spirits’; ‘I have felt calm and relaxed’; ‘I have felt active and vigorous’; ‘I woke up feeling fresh and rested’; and ‘My daily life has been filled with things that interest me’. The respondent is asked to rate how well each of the five statements applies to them when considering the last 14 days. Each of the five items is scored from 5 (all of the time) to 0 (none of the time). The raw scores range from 0 (worst thinkable well-being) to 25 (best thinkable well-being). A score of < 13 is indicative of depression in Japanese patients with diabetes [22]. As the present study focuses on perceptions of optimal subjective well-being, an alternative classification was used. The top quartile (scores of 16–25) was classified as optimal well-being and the remaining quartiles (scores of 0–15) were classified as non-optimal well-being, based on a previous study [13] in which the top quintile was classified as optimal well-being. Cronbach’s *α* of the overall between-factors score for the WHO-5 was 0.91. The correlation coefficients of the WHO-5 scores of 2016 and 2017 was 0.75, suggesting that the stability of the Japanese version of the WHO-5 is good.

### Measurement of lifestyle behaviours

Lifestyle behaviour was assessed using an original questionnaire as well as a ‘specific health check-up’ questionnaire. Specific health check-ups for preventing metabolic syndrome, conducted every year under the Act of Assurance of Medical Care for Elderly People, included 22,586,005 individuals who were medical expense insurance members aged 40–75 years in 2010 [23]. Most questionnaires in this study complied with specific health check-up surveys. Dietary habits were examined based on the following items: length of lunch time (< 10 min, 10–20 min, > 20 min), preference for salty foods (more preferred, moderate, less preferred), eating vegetables (never, once per day, with every meal), subjective eating speed (quick, moderate, slow), dinner within two hours before going to sleep (yes/no), snacking after dinner over three times per week (yes/no), and skipping breakfast over three days per week (yes/no). Physical activity was examined by the following items: over 30 min of intensive exercise more than twice per week (yes/no) and walking for over one hour every day (yes/no). Smoking status (current, never), alcohol consumption (< 22 g, 22–43 g, 44–65 g, > 66 g; ethanol conversion/day), sleep quality (good, poor), and subjective stress condition were also assessed.

Additionally, to investigate the association between lifestyle changes and optimal well-being, change in dietary habits (‘dinner within two hours before going to sleep’ and ‘snacking after dinner over three times per week’) and physical activity (‘over 30 min of intensive exercise more than twice per week’ and ‘walking for over one hour every day’) from 2016 to 2017 were measured by categorising responses into the following: [1] ‘maintaining bad habits’ (‘yes’ for both dietary habits items in 2016 and 2017; ‘no’ for both physical activity items in 2016 and 2017, 2) ‘worsening’ (‘no’ in 2016 and ‘yes’ in 2017 for dietary habits items; ‘yes’ in 2016 and ‘no’ in 2017 for physical activity items) [3]; ‘improving’ (‘yes’ in 2016 and ‘no’ in 2017 for dietary habits items; ‘no’ in 2016 and ‘yes’ in 2017 for physical activity items); and [4] ‘maintaining good habits’ (‘no’ for both dietary habits items in 2016 and 2017; ‘yes’ for both physical activity items in 2016 and 2017).

### Statistical analysis

Raw WHO-5 scores are presented as mean ± standard deviation (SD). Group comparisons were analysed by the Mann-Whitney U and Tukey-Kramer tests. Logistic regression analysis was used to examine the association between lifestyle behaviour and well-being. Optimal and non-optimal well-being were used as dependent variables; each lifestyle behaviour (dietary habits, physical activity, smoking, alcohol consumption, and sleep/stress condition), change in dietary and physical activity behaviours from 2016 to 2017, age, sex, and BMI were used as independent variables (Model 1). Model 2 was also adjusted for sleep quality, in addition to age, sex, and BMI. All data were analysed using the JMP 14 statistical package (SAS Institute Japan, Tokyo, Japan). Statistical significance was defined as *p* <  0.05.

## Results

### Participant characteristics

The data of 2295 Japanese individuals was used in the analysis: mean age 49.3 ± 8.4 years, 54.3% female, mean BMI 22.67 ± 3.72, and WHO-5 score 12.71 ± 4.97. There was a significant correlation between age and WHO-5 score. The correlation coefficient was 0.1013 (*p* <  0.05).

### Comparison of WHO-5 scores and various lifestyle behaviours

There were significant differences in raw WHO-5 scores depending on the stress condition (‘Do you feel stressed?’) (Never: 16.95 ± 4.23*, *n* = 387; Sometimes: 12.94 ± 4.23, *n* = 1484; Always: 7.98 ± 4.02, *n* = 411; *p <  0.01 vs Sometimes and Always) and sleep quality (Good: 15.16 ± 4.46*, *n* = 1111; Not good: 10.43 ± 4.29, *n* = 1183; *p <  0.01 vs Not good). Further, WHO-5 scores in the normal weight (12.84 ± 4.96*, *n* = 1572) and overweight groups (12.98 ± 4.91*, *n* = 421) were significantly higher (showing better well-being) than those in the underweight group (11.58 ± 4.83, *n* = 212, *p <  0.01), although no difference was observed between the obese (12.09 ± 5.49, *n* = 90) and underweight groups. Considering these results, sleep quality and BMI, in addition to age and sex, were included as independent variables in the logistic regression analysis.

Table 1 shows the comparison of WHO-5 scores for each of the lifestyle behaviours. Regarding dietary habits, WHO-5 scores were higher in those with longer lunch time, no preference for salty foods, and who ate vegetables with every meal. Moreover, not eating dinner within two hours of going to sleep and not snacking after dinner over three times per week resulted in markedly higher WHO-5 scores. Subjective eating speed did not affect WHO-5 scores.

**Table 1:** Results of the differences between WHO-5 raw scores for each lifestyle behaviour

Lifestyle behaviours	n	WHO-5	score	***p***
		Mean	SD	
**Length of lunch time**				
< 10 min	426	11.96	4.90	
10–20 min	1527	12.80	4.90	< 0.01
> 20 minutes	323	13.24	5.31	< 0.01
vs < 10 min				
**Salty food preference**				
More preferred	193	11.75	4.93	
Moderate	1694	12.55	4.90	
Less preferred	395	13.89	5.09	< 0.01
vs more preferred and moderate				
**Eating vegetables**				
With every meal	916	13.91	5.01	< 0.01
Once/day	1286	11.98	4.75	
Never	70	10.63	5.23	
vs once/day and never				
**Subjective eating speed**				
Quick	810	12.73	5.06	
Moderate	1287	12.77	4.91	
Slow	195	12.31	5.00	Ns
**Dinner within 2 hours before sleep**				
Yes	606	12.02	4.83	
No	1687	12.96	5.00	< 0.01
vs Yes				
**Snacking after dinner over 3 times/week**				
Yes	657	12.10	4.83	
No	1638	12.97	5.01	< 0.01
vs Yes				
**Skipping breakfast over 3 days/week**				
Yes	342	11.58	5.23	
No	1953	12.92	4.90	< 0.01
vs Yes				
**Over 30 min of intensive exercise more than twice per week**				
Yes	442	14.47	4.83	< 0.01
No	1851	12.29	4.91	
vs No				
**Over one-hour walking/day**				
Yes	1002	13.09	4.95	< 0.01
No	1287	12.42	4.97	
vs No				
**Current smoking status**				
No	1950	12.88	4.87	< 0.01
Yes	345	11.77	5.40	
vs Yes				
**Alcohol consumption (ethanol conversion [g]/day)**				
< 22	1623	12.68	4.92	
22-43	417	12.79	5.07	
44-65	181	13.09	5.33	
> 66	66	12.11	4.55	Ns

*Ns* not significant

Concerning physical activity, those who engaged in over 30 min of intensive exercise more than twice per week and over one-hour walking every day showed higher WHO-5 scores. Respondents who did not smoke reported remarkably higher WHO-5 scores. In contrast, amount of alcohol consumption did not affect WHO-5 scores.

### Adjusted logistic regression analysis results

To explore the association between lifestyle behaviour and well-being, logistic regression analysis was used. When the model was adjusted for age, sex, and BMI (Table 2, Model 1), good dietary habits except eating speed were positively associated with optimal well-being.

**Table 2:** Association between optimal well-being and lifestyle behaviours

Characteristic	No. of participants with optimal WB/No. of participants	Model 1		Model 2	
		OR	95% CI	OR	95% CI
**Length of lunch time**					
< 10 min	97/426	1		1	
10–20 min	432/1527	1.36	1.05–1.75	1.27	0.97–1.67
> 20 minutes	102/323	1.63	1.17–2.28	1.47	1.03–2.11
**Salty food preference**					
More preferred	41/193	1		1	
Moderate	449/1694	1.33	0.92–1.92	1.27	0.86–1.88
Less preferred	146/395	2.03	1.35–3.05	2.10	1.35–3.25
**Eating vegetables**					
Never	13/70	1		1	
Once/day	287/1286	1.29	0.69–2.39	1.07	0.56–2.08
With every meal	331/916	2.48	1.33–4.61	1.89	0.97–6.67
**Subjective eating speed**					
Quick	236/810	1		1	
Moderate	353/1287	0.94	0.77–1.15	0.83	0.67–1.03
Slow	47/195	0.82	0.57–1.19	0.89	0.60–1.33
**Dinner within 2 hours before sleep**					
Yes	132/606	1		1	
No	505/1687	1.51	1.21–1.89	1.32	1.04–1.67
**Snacking after dinner over 3 times/week**					
Yes	152/657	1		1	
No	486/1638	1.33	1.07–1.65	1.27	1.01–1.60
**Skipping breakfast over 3 days/week**					
Yes	75/342	1		1	
No	536/1953	1.36	1.02–1.79	1.23	0.91–1.66
**Over 30 min of intensive exercise more than twice/week**					
No	461/1851	1		1	
Yes	175/442	1.82	1.45–2.27	1.58	1.24–2.01
**Over one-hour walking/day**					
No	326/1287	1		1	
Yes	309/1002	1.32	1.10–1.59	1.29	1.06–1.58
**Current smoking status**					
Yes	86/345	1		1	
No	552/1950	1.23	0.93–1.63	1.28	0.95–1.73
**Alcohol consumption (ethanol conversion [g]/day)**					
< 22	448/1623	1		1	
22-43	115/417	0.93	0.72–1.19	0.83	0.64–1.09
44-65	60/181	1.17	0.83–1.65	1.04	0.72–1.51
> 66	12/66	0.54	0.29–1.04	0.54	0.27–1.07

Model 1: adjusted for age, sex, and BMI; Model 2: adjusted for age, sex, BMI, and sleep quality

*OR* odds ratio, *CI* confidence interval, *WB* well-being

Furthermore, over 30 min of intensive exercise more than twice per week and over one-hour walking every day were positively associated with optimal well-being in Model 1 (Table 2).

These positive associations were attenuated after adjusting for sleep quality in Model 2, in addition to age, sex, and BMI. Eating vegetables and skipping breakfast over three days per week were no longer associated with optimal well-being after adjustment (Table 2, Model 2).

Although there was a significant difference in raw WHO-5 scores depending on current smoking status by a simple comparison, the difference was not significant after adjustments were made in Models 1 and 2 (Table 2).

### Effects of change in dietary habits and physical activity on optimal well-being

Regarding the association between lifestyle changes and optimal well-being, raw WHO-5 scores in 2017 were significantly higher in those who maintained good dietary and physical activity behaviours than in those who did not. Furthermore, maintaining good dietary and physical activity behaviours was positively associated with optimal well-being in Model 1; however, there was no significant association between maintaining good dietary habits and optimal well-being in Model 2 (Table 3 (A)-(D) and Table 4 (A)-(D)).

**Table 3:** Effect of changes in dietary habits on optimal well-being

A. Effect of changes in dietary habits (dinner within 2 hours before sleep) on WHO-5 scores in 2017						
**WHO-5 scores (raw score)**	**n**	**Mean**	**SD**		***p***	
Maintaining bad habits	307	11.88	4.86			
Worsening	145	12.28	4.91			
Improving	166	12.77	4.99			
Maintaining good habits	1163	12.96	5.03		*p* < 0.01	
vs maintaining bad habits						
B. Association between optimal well-being and changes in dietary habits (dinner within 2 hours before sleep)						
**Characteristic**	**No. of participants with optimal WB/No. of participants**	**Model 1**		**Model 2**		
		OR	95% CI	OR	95% CI	
Maintaining bad habits	66/307	1		1		
Worsening	30/145	0.94	0.58–1.54	0.91	0.54–1.54	
Improving	50/166	1.59	1.03–2.45	1.58	0.99–2.52	
Maintaining good habits	347/1163	1.52	1.12–2.07	1.36	0.98–1.89	
C. Effect of changes in dietary habits (snacking after dinner over 3 times/week) on WHO-5 scores in 2017						
**WHO-5 scores (raw score)**	**n**	**Mean**	**SD**		***p***	
Maintaining bad habits	341	12.06	4.87			
Worsening	164	11.95	4.83			
Improving	175	12.58	4.66			
Maintaining good habits	1105	13.04	5.09		*p* < 0.01	
vs maintaining bad habits						
D. Association between optimal well-being and changes in dietary habits (snacking after dinner over 3 times/week)						
		**No. of participants with optimal WB/No. of participants**	**Model 1**		**Model 2**	
			OR	95% CI	OR	95% CI
Maintaining bad habits		78/341	1		1	
Worsening		36/164	0.91	0.58–1.44	0.95	0.59–1.55
Improving		48/175	1.25	0.82–1.90	1.29	0.82–2.04
Maintaining good habits		333/1105	1.36	1.02–1.81	1.30	0.95–1.77

Model 1: adjusted for age, sex, and BMI; Model 2: adjusted for age, sex, BMI, and sleep quality

*OR* odds ratio, *CI* confidence interval, *WB* well-being

**Table 4:** Effect of changes in physical activity on optimal well-being

A. Effect of changes in physical activity (over 30 min of intensive exercise more than twice/week) on WHO-5 scores in 2017					
**WHO-5 scores (raw score)**	**n**	**Mean**		**SD**	***p***
Maintaining bad habits	1340	12.24		4.93	
Worsening	77	13.38		5.09	
Improving	126	13.57		5.04	*p* < 0.05
Maintaining good habits	239	14.62		4.85	*p* < 0.01
vs maintaining bad habits					
B. Association between optimal well-being and changes in physical activity (over 30 min of intensive exercise more than twice/week)					
	**No. of participants with optimal WB/No. of participants**	**Model 1**		**Model 2**	
		OR	95% CI	OR	95% CI
Maintaining bad habits	329/1340	1		1	
Worsening	27/77	1.56	0.96–2.55	1.31	0.77–2.22
Improving	36/126	1.18	0.78–1.78	1.00	0.64–1.55
Maintaining good habits	101/239	2.05	1.53–2.75	1.71	1.24–2.36
C. Effect of changes in physical activity (over one-hour walking/day) on WHO-5 scores in 2017					
**WHO-5 scores (raw score)**	**n**	**Mean**		**SD**	**p**
Maintaining bad habits	772	12.28		4.91	
Worsening	230	12.77		5.11	
Improving	231	12.76		5.06	
Maintaining good habits	546	13.27		5.01	*p* < 0.05
vs maintaining bad habits					
D. Association between optimal well-being and changes in physical activity (over one-hour walking/day)					
**Characteristic**	**No. of participants with optimal WB/No. of participants**	**Model 1**		**Model 2**	
		OR	95% CI	OR	95% CI
Maintaining bad habits	183/772	1		1	
Worsening	68/230	1.44	1.03–2.01	1.45	1.01–2.08
Improving	68/231	1.39	1.00–1.94	1.31	0.91–1.87
Maintaining good habits	174/546	1.53	1.19–1.96	1.46	1.12–1.91

Model 1: adjusted for age, sex, and BMI; Model 2: adjusted for age, sex, BMI, and sleep quality

*OR* odds ratio, *CI* confidence interval, *WB* well-being

## Discussion

There was a significant difference in WHO-5 scores between those who responded ‘always stressed’ and those who responded ‘never stressed’, suggesting that WHO-5 scores are an appropriate indicator of well-being in this study population. Additionally, there was a significant difference in WHO-5 scores depending on sleep quality, which is not surprising as the WHO-5 includes a sleep-related item. A previous study reported a significant impairment in the WHO-5 total score of shift-workers compared to that of day-workers, demonstrating the importance of sleep for well-being [24].

Furthermore, the WHO-5 scores of the normal and overweight groups were significantly higher than those of the underweight group, although no difference was observed between the obese and underweight groups. According to a previous study, women with general obesity were less likely to have depressive symptoms in the low-stress group and women with abdominal obesity were 60% less likely to have depressive symptoms [25]. Although the association between obesity and psychological distress is not yet clearly understood, the overweight and normal weight individuals reported better well-being among our study population.

Regarding dietary habits, the WHO-5 scores of those with longer lunch time and who did not prefer salty food were significantly higher; these behaviours were associated with optimal well-being in Model 2 in the present study. It was reported among both sexes that daily intake of well-balanced meals and milk products were related to the absence of depressive symptoms, and avoidance of excess salt and fat was related to the absence of depressive symptoms among men [26]. Moreover, greater perceived stress was associated with lower fruit, vegetable, and protein intake, greater consumption of salty snacks, and lower physical activity levels [27, 28], further supporting the importance of dietary habits for mental health.

Additionally, those who did not have dinner within two hours before sleep and did not snack after dinner over three times per week showed higher WHO-5 scores; these behaviours were associated with optimal well-being after adjusting for age, sex, BMI, and sleep quality. This is consistent with a previous study indicating that among those with night-eating syndrome, the Beck Depression Inventory score was indicative of moderate depression in 18.5% of cases and of severe depression in 44.4%, suggesting an association between night-eating behaviour and psychological distress [29]. Moreover, the positive association between maintaining these healthy dietary habits for two years and optimal well-being indicates the importance of maintaining such behaviours, although the significance of this association diminished after adjusting for sleep quality, in addition to age, sex, and BMI.

Further, the WHO-5 scores of those with over 30 min of intensive exercise more than twice per week and over one-hour walking every day were significantly higher and these behaviours were positively associated with optimal well-being after adjusting for age, sex, BMI, and sleep quality. These findings are consistent with previous studies reporting an association between physical activity and increased self-rated health [30], increased happiness [31], and lower anxiety [32].

Moreover, in a multi-level mixed effects model, more frequent physical activity and sport participation were both found to independently contribute to greater well-being and lower levels of anxiety and depressive symptoms in both sexes [33]. Furthermore, in adjusted isotemporal models, a 30 min increase in light activity per day was associated with a significant decrease in anxiety symptom levels and a significant increase in well-being levels [34]. The present results indicating that maintaining the studied physical activity behaviours over the year of study was associated with optimal well-being extend the findings of these previous studies. Additionally, the positive association between maintaining physical activity behaviours over the study year and optimal well-being even after adjusting for sleep quality in Model 2 suggests that it plays a more significant role than maintaining good dietary habits.

Regarding alcohol consumption, it was not associated with well-being. Not being a current smoker, although associated with well-being in the unadjusted analysis, was not significantly associated with optimal well-being in the adjusted analysis. These results are consistent with data from a previous study showing the lack of association between excellent well-being and lower use of alcohol and smoking [13]. However, further detailed investigation involving data on past smokers is necessary to determine how smoking cessation affects well-being.

This study has several limitations. First, its primarily cross-sectional nature does not allow inference of causality. Second, participants were recruited from a single healthcare centre and the mean age of the participants was around 50 years, possibly limiting the generalisability of these findings. Third, we could not enquire about people’s socioeconomic status, education, occupation, income, or wealth at the time of the health check for ethical reasons. This resulted in the possibility that we may have missed confounding socioeconomic factors. Further, because the participants could afford to attend the health check-ups, it is possible that the present group, with an average age of 50, had a high level of motivation for health awareness and maintenance, which may have affected the results. Fourth, because data on dietary and physical activity behaviours were collected using a self-reported questionnaire using ‘yes’ or ‘no’ responses, we could not verify whether the target factors were correctly captured. In other words, the answers were subjective and not objective. Moreover, the classification of well-being was not based on established scoring criteria, although this method was adopted from a previous study [13]. Future research should examine the association between lifestyle behaviours and positive well-being in a population with greater sociodemographic diversity using quantitative measures.

## Conclusion

Not only currently practising good dietary and physical activity behaviours but also maintaining such behaviours over time is associated with optimal well-being. Maintaining good lifestyle behaviours, particularly regarding physical activity, could improve people’s well-being. Primary care physicians need to be aware of this aspect and take it into account during medical consultations.

## Data Availability

The datasets used and/or analysed during the current study are available from the corresponding author on reasonable request.

## Abbreviations

CI: Confidence interval
Ns: Not significant
OR: Odds ratio
SD: Standard deviation
WHO-5: The 5-item World Health Organization Well-Being Index
